# Aortic valve neocuspidization for a low-birth-weight neonate with severe aortic stenosis with regurgitation

**DOI:** 10.1016/j.xjtc.2026.102293

**Published:** 2026-02-25

**Authors:** Masaya Nakamizo, Yuji Tominaga, Masashi Takeshita, Takuji Watanabe, Keisuke Shibagaki, Takako Toda, Kenichi Kurosaki, Shigemitsu Iwai

**Affiliations:** a Department of Pediatric Cardiovascular Surgery, National Cerebral and Cardiovascular Center, Suita, Osaka, Japan; b Department of Pediatric Cardiology, National Cerebral and Cardiovascular Center, Suita, Osaka, Japan

**Figure:**
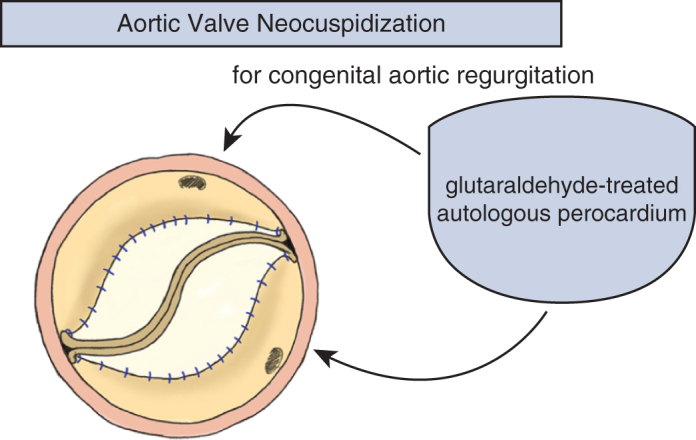
Image of bicuspid neocuspidization for a small annulus.

**Central Message**

Aortic valve neocuspidization allowed us to avoid the Ross procedure during the neonatal period and provided a safe bridge for future interventions.

Surgical intervention for congenital aortic stenosis with regurgitation (ASR) during the neonatal period is challenging because of the small annular size and severe leaflet degeneration. Although the Ross procedure offers favorable long-term outcomes in pediatric patients, its application in neonates or low-birth-weight infants carries early mortality risk. Staged strategy consisting of initial valve repair to delay the Ross procedure until sufficient weight gain is achieved has been proposed.^1^ Aortic valve neocuspidization has recently been introduced in pediatric patients with severely dysplastic valve cusps.

We describe the case of a low-birth-weight neonate with critical ASR who was successfully treated using a staged strategy comprising initial neocuspidization followed by planned Ross procedure after weight gain. Informed consent for publication was obtained from the patient's legal guardians. This case report was approved by the Institutional Review Board of the National Cerebral and Cardiovascular Center (Approval No.: R19092; approval date, December 20, 2019).

## Case Report

**Figure 1:**
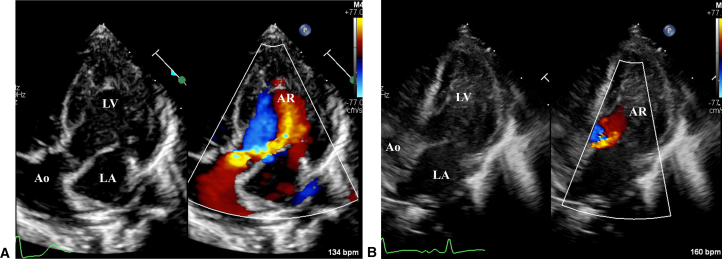
A, Preoperative transthoracic echocardiography showing severe aortic regurgitation. B, Postneocuspidization transthoracic echocardiography showing mild aortic regurgitation. *LV*, Left ventricle; *LA*, left atrium; *Ao*, aorta; *AR*, aortic regurgitation.

The fetus was diagnosed with severe AR on fetal echocardiography, with concerns of hydrops fetalis, and mother was transferred to our hospital. A female infant weighing 2110 g was delivered at 38 weeks’ gestation. Postnatal transthoracic echocardiography revealed severe aortic regurgitation and moderate aortic stenosis (Figure 1, *A*). During the early neonatal period, she presented with heart failure symptoms, progressive left ventricular dilatation (left ventricular end-diastolic diameter of 22.1 mm; *z* score, 3.5) and insufficient weight gain. She underwent aortic valve neocuspidization at 19 days of age and 2106 g weight.

**Figure 2:**
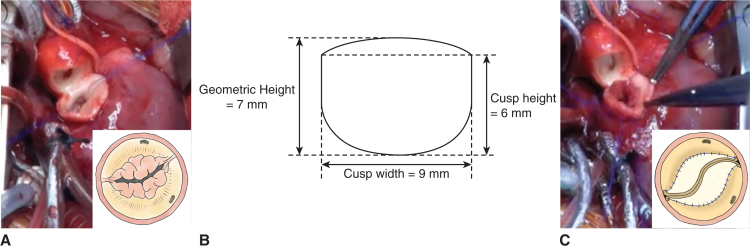
A, Operative picture showing the native aortic valve. B, Neocusp design. C, Neocuspidized aortic valve.

After cardiac arrest with cold blood cardioplegia, a transverse aortotomy was performed 5 mm above the sinotubular junction. The native aortic valve was bicuspid with fusion of the right and noncoronary cusps. The valve leaflets were significantly degenerated and thickened, with nodular changes precluding aortic valvuloplasty (Figure 2, *A*). Given the small aortic annulus diameter (6 mm), we performed neocuspidization by adopting a 180° bicuspid configuration. A new commissure was established directly opposite the native commissure between the right and left coronary cusps.

Neocusps were fashioned from autologous pericardium and treated with 0.6% glutaraldehyde for 3 minutes, based on concerns regarding the balance between leaflet stability and excessive stiffness. As shown in Figure 2, *B*, the valve height and width of the neocusp were the annulus diameter and half of the annulus circumference, respectively. Effective neocusp height was determined based on normal values reported in the literature.^2^

All native leaflets were excised at their base, and each neocusp was sutured using a running 7-0 monofilament suture, starting from the commissure between the right and left coronary cusps, with trimming to ensure an adequate coaptation height (Figure 2, *C*). After closing the aortotomy in 2 layers, deairing was performed, and the aorta was declamped. The surgical procedure is demonstrated in Video 1.

Postoperative echocardiography revealed a mild residual ASR (Figure 1, *B*), and the patient steadily gained weight and ASR severity gradually worsened. At age 7 months and weighing 5.7 kg, she underwent an uneventful Ross procedure. At the time of this report, patient remained clinically well with no complications observed during the 2 years of outpatient follow-up.

## Discussion

Several advantages of valve repair have been reported in the context of pediatric aortic valve surgery, and principles of the surgical approach have been proposed.^3^ However, aortic valve repair for neonatal ASR is often technically challenging because of severely degenerated valve leaflets, necessitating complex techniques including patch materials usage. This difficulty further increases when the procedure is performed on a small aortic annulus.

Aortic valve neocuspidization is a reproducible surgical technique that is less dependent on the native valve leaflets quality and avoids difficulties associated with preserving native tissue. Neocuspidization in pediatric patients has demonstrated favorable short-term outcomes,^4^ and a previous report^5^ showed a lower risk of reoperation than aortic valve plasty with patch augmentation, making it a valuable option.

We performed bicuspidization owing to a small aortic annulus. Constructing 3 cusps in such a limited space is technically more difficult than bicuspidization and may increase postoperative stenosis risk due to reduced orifice area. A previous study^E1^ showed favorable outcomes in valve repair for bicuspidization over tricuspidization, supporting the validity of this approach.

In our case, the procedure was successfully performed in a low-birth-weight neonate (2.1 kg) with a small annulus (6 mm). Bicuspid neocuspidization allowed us to avoid the Ross procedure during the neonatal period and provided a safe bridge for future interventions.

## Conflict of Interest Statement

The authors reported no conflicts of interest.

The *Journal* policy requires editors and reviewers to disclose conflicts of interest and to decline handling or reviewing manuscripts for which they may have a conflict of interest. The editors and reviewers of this article have no conflicts of interest.

## Supplementary Data

Video 1 Surgical video demonstrating aortic valve neocuspidization for the low-birth-weight neonate. Video available at: https://www.jtcvs.org/article/S2666-2507(26)00100-8/fulltext.
