# Supplementary figures and images for: Aortic valve neocuspidization for a low-birth-weight neonate with severe aortic stenosis with regurgitation
Source: JTCVS Tech. 2026 Feb 25;37:102293. doi: 10.1016/j.xjtc.2026.102293 (PMC13261157; doi:10.1016/j.xjtc.2026.102293)

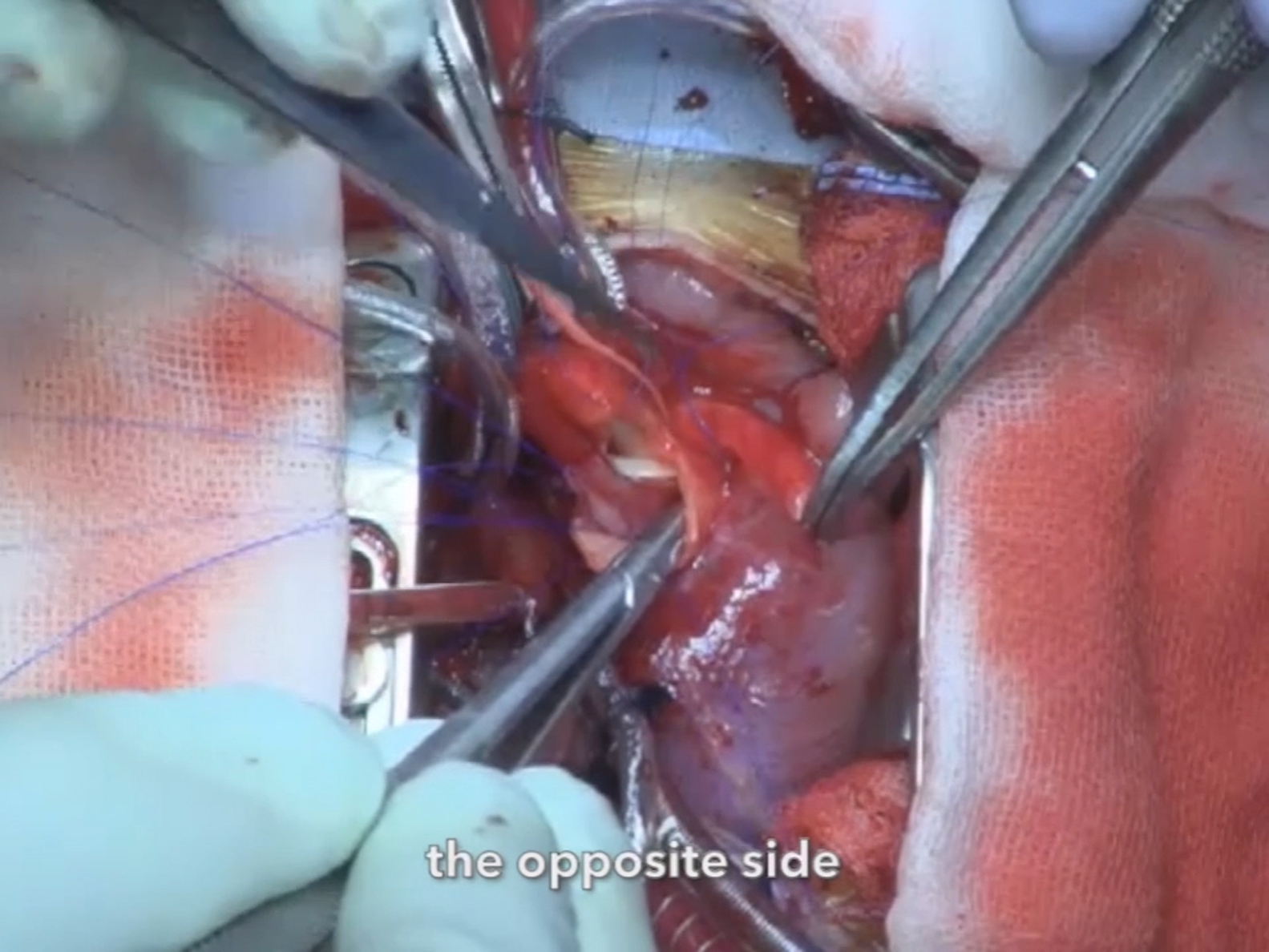

Supplement: Video 1 — Surgical video demonstrating aortic valve neocuspidization for the low-birth-weight neonate. Video available at: https://www.jtcvs.org/article/S2666-2507(26)00100-8/fulltext. [file fx2.jpg]
